# Current State of Modern Biotechnological-Based* Aeromonas hydrophila* Vaccines for Aquaculture: A Systematic Review

**DOI:** 10.1155/2019/3768948

**Published:** 2019-07-29

**Authors:** Alexanda Mzula, Philemon N. Wambura, Robinson H. Mdegela, Gabriel M. Shirima

**Affiliations:** ^1^Department of Global Health and Biomedical Sciences, School of Life Science and Bioengineering, Nelson Mandela African Institution of Science and Technology, Arusha, Tanzania; ^2^College of Veterinary Medicine and Biomedical Sciences, Sokoine University of Agriculture, Morogoro, Tanzania; ^3^National Ranching Company (NARCO), Ministry of Livestock and Fisheries Development, Tanzania

## Abstract

This systematic review describes what “the cutting edge vaccines for* Aeromonas hydrophila* are”. The focus is on types of high tech biotechnological based vaccines, target gene or antigen in developing these vaccines, and challenge model fish species used in vaccines efficacy testing. Vaccines delivery methods, immune response, and their efficacy, adjuvant or carrier systems used, and the overall experimental setup or design of the vaccines under investigation are also described. The search for the original papers published between 2009 and 2018 was conducted in June of 2018, using the PubMed and Google scholar electronic database. Twenty-three (23/4386) studies were included in the final assembly using PRISMA guidelines (Protocol not registered). Recombinant protein vaccines were the highly experimented type of the modern biotechnological based vaccines identified in the selected studies (16/23; 70%). Outer membrane proteins (OMPs) of different *β*-barrels were shown to be a potential antigenic entity for* A. hydrophila* vaccines (57%). Intraperitoneal route with conventional carries or adjuvants was the highly applied delivery system while very few studies used herbal based vaccine adjuvants and nanomaterial as a vaccine carrier. Variation was observed in terms of protection levels in the selected studies. The experimental designs partly contributed to the observed variation. Therefore, recombinant vaccines that use new carrier system technologies and delivered through oral route in feeds would have been of great value for use in the prevention and control of* A. hydrophila *infections in fish. Despite the usefulness as academic tools to identify what is important in pathogenicity of the etiological agent to the host fish, these vaccines are only economically viable in very high-value animals. Therefore, if vaccination is a good option for* A. hydrophila* group, then simple autogenous vaccines based on accurate typing and evidence-based definition of the epidemiological unit for their use would be the most viable approach in terms of both efficacy and economic feasibility especially in low and middle-income countries (LMIC).

## 1. Introduction

Aquaculture has been stipulated to play a prodigious role in food security after fisheries. It serves as a source of income at the household level as well as at the national level in developed and developing countries [1]. Due to great demand for fish protein, the aquaculture sector attracted great attention and it is a fast-growing agricultural sector [2]. To maximize yield, the culture system has become in practice more intensively and hence among others, fish diseases have started to become a disaster especially in countries where aquaculture is operational [3]. Bacterial diseases are the most leading causes of fish mortality in aquaculture. Despite the known contributions of other species of the genus* Aeromonas* in causing diseases in fish,* A. hydrophila* is the main cause of disease outbreaks in freshwater farmed fish contributing to food insecurity and economic loss worldwide [4–6]. The bacterium causes various diseases in fish named as haemorrhagic septicaemia, dropsy, epizootic ulcerative syndrome, haemorrhagic enteritis, and red body disease [7, 8]. Aeromonads diseases in fish farms are accelerated by several factors including variations in physical-chemical parameters of pond water.

Despite the fact that vaccination represents the most effective strategy to prevent diseases in the aquaculture industry [30], commercial vaccines for* A. hydrophila* in fish have been a challenge [6]. This gram-negative rod-shaped bacterium of the family* Aeromonadaceae* causes several signs of ill health including tail and skin rot and fatal haemorrhagic septicaemias in several fish species [31]. Owing to its nature of being ubiquitous of the aquatic environment, this bacterium has become a thought-provoking pathogen of fish which requires maximum pond management practices and biosecurity measures to control it [14]. Although the application of antibiotics is not healthy for fish consumers, still its effectiveness is questionable because of the delay of disease diagnosis and increase in antibiotic resistance, which has been shown by the bacterium worldwide [32].

One of the problems that limit the development of commercial* A. hydrophila* vaccines is strain diversity [33] and failure of the vaccine to confer protection to heterologous strains [34]. However, an effort has been made to develop vaccines in different regions worldwide, initially focusing on inactivated products and live attenuated organisms. Following advancement made in molecular biology, biotechnology, vaccine immunology, and reverse vaccinology, new high tech vaccines are being developed and experimentally tested against* A. hydrophila* in different fish species. Therefore, this systematic review describes what the current knowledge in* A. hydrophila* vaccines development is. The focus is on types of high tech biotechnological based vaccines, target gene or antigen in developing these vaccines, and challenge model fish species used in vaccines efficacy testing. Vaccines delivery methods, immune response, and their efficacy, adjuvant or carrier systems used, and the overall experimental setup or design of the vaccines under investigation are also described. The rationale for reviewing these vaccines to this specific pathogen was that, in past years, vaccination has significantly contributed to minimizing the disease burden in the aquaculture system using conventional vaccines. However, the use of modern biotechnological vaccines has the potential to fill the gap between vaccine efficiency and increased demand. To the best of our knowledge, this systematic review is of its own kind that looked at these studies on modern biotechnological vaccines against* A. hydrophila* as a whole.

## 2. Methodology

### 2.1. Searching Strategy and Selection Criteria

The following search words, “DNA” or “Recombinant” or “Subunit” and “Vaccines “and* A. hydrophila* and in fish, were used in combination with Boolean operator search as described by Boell and Cecez-Kecmanovic [35, 36] to identify articles to be included in this systematic review. The search for the papers was conducted in June of 2018, using the PubMed and Google scholar electronic database. Hand searching for a bibliography of included studies to identify any other potential articles was also done. The following criteria were used for including a source in the study: publications had to be in English; the publication date had to be between 2009 and 2018; the published articles had to be an original article; experiments on the immunological responses and efficacy had to be done in fish and the delivery methods information of the vaccine under test had to be available; the studies had to focus on fish vaccines against* A. hydrophila* leading to the rejection of all or most fish vaccines related to other bacterial species. This review is written following the PRISMA method; however, this protocol was not registered with PRISMA.

### 2.2. Data and Information Collection Process

All publications which met the inclusion criteria were entered in Mandalay reference manager and publications were ordered by primary authors. Information was extracted from each included study on type of modern biotechnological based* A. hydrophila* vaccines in fish (e.g., plasmid DNA vaccine, recombinant protein vaccine), target gene or antigen in developing* A. hydrophila* vaccine in fish, working mechanism in relation to immune responses and protection, challenge model fish species used in vaccine efficacy testing, delivery methods, and adjuvants and/or carrier system employed.

## 3. Results and Discussion

### 3.1. Description of Literature Search Outcome

**Figure 1:**
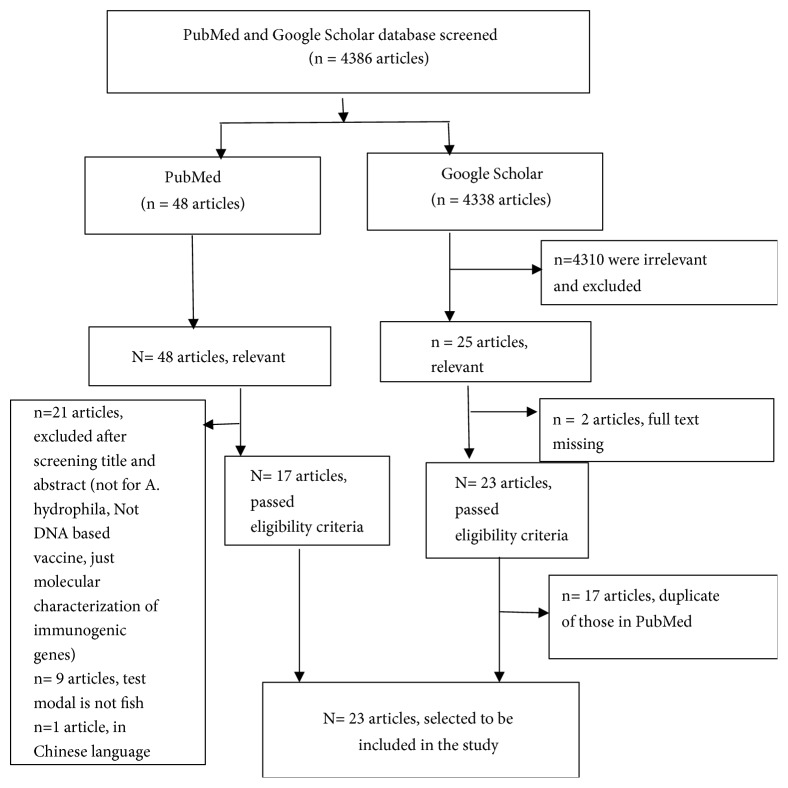
A flow diagram showing inclusion and exclusion criteria of selected studies for this systematic review.

In literature searching, 4386 articles were identified from both PubMed and Google scholar, of which 4310 were irrelevant and excluded just after screening the manuscript titles while 73 articles (Figure 1) seemed to be relevant to the study in question. A further detailed assessment of titles and abstract revealed 21 articles (n=21) that were not for* A. hydrophila*, not either DNA based or recombinant vaccine but just molecular characterization of immunogenic genes and therefore were excluded from this review. On the other hand, some articles were not included in the analysis as they missed inclusion criteria or other overbearing reasons such as duplicate articles (n = 17); articles lacking full text (n = 2); articles in which the vaccine test model was not fish (n = 9); and 1 article in which the text was in Chinese language. Of the articles in which the test model was not fish, 5 used mice as a test model but the vaccine is anticipated to protect fish and the test model of 4 articles were mice but the vaccine aimed to protect humans.

The most important findings and information revealed and extracted from eligible articles (n = 23) used in this systematic review were types of modern biotechnological vaccines against* A. hydrophila* in fish; target gene or antigen used in developing* A. hydrophila* vaccine in fish; working mechanism in relation to immune responses and efficacy evaluation; challenge model fish species involved in vaccine efficacy testing (Table 1), delivery methods used and adjuvant/ carrier system employed. Most of the selected articles from this review emanated from the findings obtained from China (12/23; 52%) followed by India (5/23; 22%) (Table 2). The criteria used to exclude some studies from the review have been shown in Figure 1.

### 3.2. Types of Modern Biotechnological Vaccines against* A. hydrophila* in Fish

It is evident that whole organism vaccines (killed and attenuated vaccines) showed better advantages than other types of vaccines. Attenuated vaccines, for example, have great potential in aquaculture in the sense that they provide a simulation model of infection and the vaccine strain could spread to a nonvaccinated fish population over a prolonged period of time. Furthermore, live attenuated vaccines have the advantage that they stimulate humoral and cellular immunity significantly in fish. But the matter of fact is that not all these vaccines completely prevent disease and in addition have safety concerns [37], a time-consuming process, which delays the timely development of vaccines against emerging and reemerging pathogens of fish. Therefore, novel approaches through advances made in genetics, biotechnology, immunology, and molecular biology [38–40] were needed for discovering newer types of effective vaccines in the aquaculture field.

**Figure 2:**
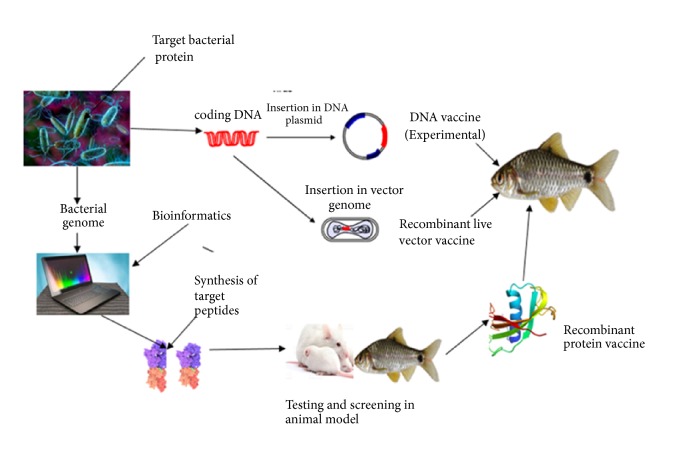
Schematic representation of types of modern biotechnological based* A. hydrophila* vaccines development employed in the selected studies.

From the reviewed articles, advances in molecular biology, biotechnology, and reverse vaccinology have enabled the development of different types of* A. hydrophila* vaccines which have recently been experimentally tested in fish. They include subunit vaccines, plasmid DNA vaccines, the recombinant live vector vaccines, and recombinant protein vaccines of which some approaches towards their developments have been shown in Figure 2. Three articles (n= 23) worked on DNA vaccines and only one article tested the recombinant live vectored vaccine (n=23) (Table 1).

DNA vaccinations against a wide range of pathogens have been investigated in various fish species especially against viral diseases but limited in bacterial diseases. Pridgeon and Klesius [28, 29] reported a high protective vaccine efficacy of 100% for their DNA vaccine delivered through intraperitoneal injection in channel catfish 2 days post injection while Liu et al. [27] on the other hand observed a relatively lower protective vaccine efficacy of about 68.9% for the DNA vaccine delivered through intramuscular injection. In spite of having several advantages such as conferring immediate, safe, and durable protection against several viral diseases such as infectious hematopoietic necrosis virus (IHNV) [5, 41] in farmed fish, this type of vaccine seemed to be less adopted in bacterial diseases and especially in controlling diseases caused by* A. hydrophila* in farmed fish. Among others, one reason given by researchers was bacteria having genes involved in the production of carbohydrates and highly glycosylated proteins of which transcription and production of plasmid DNA encoding these genes are not feasible but only possible for nonglycosylated proteins [42]. Thus DNA vaccination could not probably be a good alternative substitute for the more traditional polysaccharide containing vaccines in triggering immune responses against microbes that have an outer membrane made of, for example, lipopolysaccharides [43]. The reported possibilities of developing myositis upon intramuscular injection of plasmid DNA (pDNA) are another challenge limiting its use against bacterial infection in fish.

In this review, it has been observed that only one and indeed very few studies focused on experimenting on recombinant live vectored vaccines against* A. hydrophila* in fish. The study utilised nonpathogenic recombinant* Lactococcus lactis* to carry an aerolysin gene from* A. hydrophila*. Live vaccines, be it attenuated pathogens or microbial vectors carrying epitopes of the pathogen, always promote a potent immune response as it mimics natural infections and has intrinsic adjuvant properties than nonreplicating products [37]. However, as it has been explained by Vaughan et al. [44] that immunization with such vaccines unavoidably infers the release of recombinant organisms into the surrounding environment thus based on European Union (EU) and other guidelines, such organisms are pigeonholed as genetically modified organisms (GMO), limiting their potential utilisation and this could be the reason of very few publications of this kind of vaccines experimented against* A. hydrophila* in fish.

Recombinant protein vaccines seem to take a wide coverage in controlling most of the bacterial diseases in fish. This is depicted by a number of studies experimenting on recombinant protein vaccines against* A. hydrophila* diseases in fish, as 70% of the articles (16/23) analysed in this systematic review reported experimental findings of recombinant protein vaccines against* A. hydrophila*. These vaccines are prepared by taking only the immunogenic regions of a pathogen and insert it in an expression host that expresses the protein on a large scale and then later the protein is purified as a vaccine [45]. Initially, the development of this type vaccine was a bit tedious especially in the characterization of an immunogenic component of the pathogen, but following advancement in reverse vaccinology; vaccine development can take one to two years. In addition to quick development, vaccine safety is guaranteed upon the usage of safe and appropriate vaccine delivery systems and adjuvants. It is because of these reasons and many other vaccinologists have put the effort into experimenting on this type of vaccine against* A. hydrophila* in different fish species.

As advocated by Dalmo [46], we also agree that all of these vaccine development strategies have merits and demerits, and their use will depend on nature of the mechanisms of infection of the particular pathogen and respective immune response required for protection.

### 3.3. Target Gene or Antigen in Developing A. hydrophila Vaccines in Fish

It has been observed that 57% of the experimental vaccines for* A. hydrophila *in the selected studies targeted the outer membrane proteins of different *β*-barrels as the potential antigenic entity while others used lipopolysaccharide (LPS), aerolysin genes, N-acyl homoserine 1 lactonase, glycoprotein, recombinant S-layer protein, G-protein coupled receptor 18 (GPR18), and apolipoprotein (Table 2).

Outer membrane proteins (OMPs) serve as an interface between the host and the pathogen. The use of OMPs in several studies for* A. hydrophila* recombinant and subunit vaccines design to develop vaccine candidates because of their association with pathogenesis, adherence, and the invasion of the pathogen to the host fish is well recognised [18]. The OMPs serve as an interface between the pathogen and the immune cells. Therefore, the use of OMPs in most of the experimental vaccines in the selected studies may have been driven by the reported protection success of few OMPs from various bacterial species in fish [6].

Although there are other antigenic targets that have been proved to confer immunity in fish against* A. hydrophila*, it is our filling that the increasingly use of recombinant OMPs as vaccines candidates for* A. hydrophila* in fish came after realisation of conserved nature of antigenic determinants that induce specific immune system in the host fish, providing solution against the existing antigenic strains diversity hurdle in development of effective commercial vaccines against the bacterium (Lutwyche et al. 1995) [6]. Therefore in addition to the reported attributes of inducing specific antibodies, inhibiting bacterial colonization, and inducing cell-mediated immunity, its conserved nature provides cross-protection against several bacterial strains and species in fish.

Although most of the OMPs of different *β*-barrels have been shown to be potential candidate vaccines against* A. hydrophila, *we apparently assume that the synergistic immune response would have been reached when these were combined and therefore further research should be directed on testing the combination of these OMPs barrels in recombinant protein vaccine formulation.

### 3.4. Model Fish Species Used in Vaccine Efficacy Testing

The use of grass carp as a fish model in testing the experimented* A. hydrophila *vaccines has been observed in four studies (4/23; 17%; Table 2). Similarly, four studies (4/23; 17%) reported having used Channel Catfish as a vaccine testing model. Rohu has been used in three studies (3/23; 13%) while only one study (1/23; 4%) tested the vaccine efficacy on tilapia. Twelve studies (12/23; 52%) used different fish model species. Fish is a heterogeneous cluster of organisms that include the agnathans (lampreys and myxines), condryctians (sharks and rays), and teleosteans (bony fish) [47]. Despite the fact that vaccines in aquaculture are specific to fish species, the variability observed in experimental challenge fish species used in vaccine trials may have been also backed by the most common farmed fish in respective location or region the study has been carried out. Most of the studies included in this review originated from Asia (17/23, 74%) where the common carp and grass carp are cultured in many countries in Asia and Europe [48]. As Mitchell [49] put it, that limited number of vaccines for tilapia is available today making this market segment of tilapia vaccines relatively novel.

### 3.5. Vaccines Delivery Methods

Vaccine administration in fish is done through different routes such as oral administration, intramuscularly, intraperitoneal injection, and through immersion. In the selected studies, the administration of experimented* A. hydrophila* vaccines by intraperitoneal injection has been reported by eighteen studies (18/23; 78%; Table 1) while only one study used bath immersion (1/24; 4%) to vaccinate the model fish. While efforts are made by researchers to improve vaccine carriers in a way that can accommodate mass vaccination of fish, vaccine delivery for most of the bacterial fish vaccines through intraperitoneal injection and for DNA vaccines through intramuscular injection has currently been common in fish.

The increased number of studies in using intraperitoneal injection to deliver the vaccines has been accelerated on the truth that the method gives high protection compared to other delivery systems (Table 2). As it was pointed out by Plant and LaPatra [50], the challenges with this delivery method are that they pose stress to fish, labour intensive and therefore costly and it is suitable for large size fish. Contrary to the injection method, dip and bath immersion is applied to vaccinate fish of all sizes using a different concentration of vaccines. However, this method is pointed to have low vaccine protection of which scientists proved to be caused by poor vaccine antigen uptake through skin and gills. It is just in 2002 where Nakanishi et al. [51] reported high protection of a vaccine against* Streptococcus iniae* in rainbow trout (*Oncorhynchus mykiss*) using a novel approach, a skin puncture followed by immersion delivery system. Nevertheless, we concede with animal welfare activist and other scientists that the method increases stress to fish more than that induced during injection method.

Oral administration is another method of vaccine delivery useful for mass vaccination of fish, and it is normally employed through feeds. In this study, only 3 articles assessed their vaccines protection status using oral administration. Anuradha et al. [14] reported live recombinant vaccine protection of 70 to 100% using Fraund's adjuvant as vaccine carrier while Dubey et al. [26] on the other hand assessed the recombinant protein vaccine using nanoparticles, that is, PGLA reporting protection of 37 to 80% through the same route. Furthermore, findings from other various scientists have revealed that naked antigens are prone to degradation in the foregut of the fish due to the acidic environment before reaching the hindgut where adherence and immune responses are elicited [52]. The inactivated and unencapsulated vaccines seem to be highly affected by the situation compared to live vaccines. It is also well documented that oral vaccine administration does not give reliable protection because of inconsistency in vaccine uptake by fish. We, therefore, emphasize working on targeted delivery strategies which are being used for oral vaccine development in humans and other animal species to be used extensively in vaccines against* A. hydrophila* in fish.

### 3.6. Adjuvant/Vaccine Carrier System

An immunologic adjuvant is applied to accelerate, prolong, or enhance antigen-specific immune response when combined with specific antigens [53]. Search for safer and potent vaccine adjuvants and carrier system has resulted in the formulation of antigen into different carrier systems from those of historical solution form to modern adjuvants and carrier system in particulate form. These adjuvants and carrier systems range from those of chemical-based to biological ones.

A number of scientists have said that chemical adjuvants have been historically used to enhance the efficacy of vaccines in humans, animals, and fish [54]. This is in agreement with what has been revealed in the selected studies, where Montanide adjuvants, Freund's adjuvants, and other conventional chemical adjuvants (4/23; 17% each) are the leading carrier systems used in an experimental vaccine against* A. hydrophila* in fish. Despite the reported efficiency, we concede to those who say that the conventional chemical adjuvants and vaccine carriers also produce adverse effects to the host such as chronic peritonitis, adhesions, and granulomas in extreme conditions [6, 55, 56].

Due to that, the search for better carrier systems that provide improved vaccine efficacy especially in new generation vaccines such as subunit, DNA, and recombinant protein vaccines was instigated. The use of biological adjuvant such as molecular adjuvants, i.e., plasmid-encoded cytokine adjuvants in DNA vaccines [57], herbal based adjuvants, i.e., Asparagus racemosus extracts [11], nanotubes, and nanoparticles have gained special attention in human and animal vaccines [26]. However, as it has been observed in this review of modern biotechnological based* A. hydrophila* vaccines (Table 1), the application of these new carrier systems is nearly inattentive in fish. Only two studies used the biological based adjuvants, the* A. racemosus* extracts (Thangaviji et al. 2012) and modified herbal adjuvant [6]. Four studies reported the use of nanomaterials as vaccine carrier system for* A. hydrophila* vaccines, which includes single-walled carbon nanotubes (SWCNTs) (3/4) and poly lactic-co-glycolic acid (PLGA) nanoparticle (1/4) (Table 1).

Microencapsulation of vaccines in polymers such as chitosan, MicroMatrix™, alginates, liposome, and poly D,L-lactic-co-glycolic acid (PLGA) are the current novel approaches towards improving oral vaccines incorporated in the feed [52]. The application of biodegradable PLGA nanoparticles, for example, has attracted a lot of interest as an antigen carrier system for oral vaccines because of their ability to enhance antigen uptake and ability to allow the slow release of antigens in vivo [26] and therefore we advocate research on nanomaterial carrier systems for oral vaccines against* A. hydrophila* in fish as the use of injectable vaccines in mass vaccination of fish becomes complicated.

### 3.7. Working Mechanism in Relation to Immune Responses and Vaccine Efficacy

Vaccines work by inducing either humoral immunity or both humoral and cellular immunity. In the selected study, only four studies (4/23; 17%) reported having assessed the humoral and adaptive cellular immune response of their vaccines while the majority reported innate and antibody-mediated immunity only without the adaptive cellular immunity. This is in agreement with what was observed by Munang'andu and Evensen [58] that very few studies in fish immunology showed protection capacity of cell-mediated immunity. Although it is well known that the immune response in fish resembles that of mammals with some specific differences between them [59], assessment of the immune responses in fish is not a straightforward activity. The measurement of humoral immunity can be possible but on the other hand, cell-mediated immunity cannot be easily assessed [7]. This perhaps could be the reason why most of the studies included in this review failed to assess the adaptive cell-mediated immunity.

In line with that, the challenges in designing these new kinds of vaccine strategies to elicit the appropriate cellular immunity [45, 58] and the extracellular nature of the bacterium could be another reason of assessing the humoral immunity rather than cellular immunity. Correlate of protection (CoP) has been established for some licensed human and animal bacterial vaccines [46]. This could be an additional reason that drove researchers in the selected studies to opt to assess the antibody responses as correlate of protection without cell-mediated immunity; however a study conducted by Abdelhamed et al. [7] on recombinant* A. hydrophila* vaccine in fish revealed that antibodies responses did not correlate with the protection level while the relative percent survival (RPS) showed fish to be protected following challenge. Abdelhamed et al. [7] therefore explained this scenario by acknowledging that antibodies do not account for all of the protection and the predominance of cellular immunity over the antibodies responses cannot be undervalued.

Most of the selected studies assessed vaccine efficacy in terms of RPS, of which four studies did so without assessing vaccine immunogenicity. Nonetheless understanding the immunological mechanism of the vaccine under study is very important. Furthermore irrespective of the reported promising vaccine efficacies (in terms RPS) abridged in Table 1, it is hard to draw general conclusions because of differences in experimental design observed in the selected studies (Table 3) such as the dose, vaccine type, challenge fish model species, interval between vaccination and challenge (Table 3), route of administration (Table 2), and vaccine adjuvant effects. Most of the selected studies conducted an experimental vaccine challenges 30 days postvaccination while only 1 conducted for 120 days postvaccination.

As Johansen et al. [60] put it, we also insist that common experimental design and guidelines for specific fish species in addition to other general guidelines such as that developed by European Medicine Agency (EMA) [61] should be established. This will assist researchers to have a common understanding of protection trials of their newly developed vaccines in fish.

Therefore it is here emphasized in agreement with Mweemba and Evensen [62] that consensus should be reached on a correlate of protection based on challenge models, measures of efficacy, and immunological mechanisms of vaccine protection.

## 4. Conclusion

Recombinant vaccines that use new carrier system technologies and are delivered through oral route in feeds would have been of great value for use in the prevention and control of* A. hydrophila* infections in fish as it could support mass vaccination in a similar way it does in other bacterial diseases in fish. However, recombinant vaccines are really useful academic tools in identifying what is important in pathogenicity of the etiological agent to the host fish but are only economically viable in very high-value animals. Therefore, if it is believed that vaccination is a good option for* A. hydrophila* group, then simple autogenous vaccines based on accurate typing, diagnostics, and evidence-based definition of the epidemiological unit for their use would be the most viable approach in terms of both efficacy and economic feasibility especially in low and middle-income countries (LMIC).

## Figures and Tables

**Table 1:** Summary of vaccines and the delivery systems used in the selected studies.

Type of vaccine	Adjuvant/vaccine carrier system	Country	Reference
Subunit vaccine	-	China	[9]
Subunit vaccine	Montanide and aluminium hydroxide	Turkey	[10]
Subunit vaccine	Asparagus racemosus extracts	India	[11]
Subunit vaccine	Freund's adjuvant	China	[12]
Recombinant protein vaccine	non-mineral oil adjuvant Montanide ISA	USA	[13]
Recombinant protein vaccine	non-mineral oil adjuvant Montanide ISA 763 AVG	USA	[7]
Recombinant live vectored vaccine	Freund's adjuvant	Malaysia	[14]
Recombinant protein vaccine	-	China	[15]
Recombinant protein vaccine	PBS-mineral oil modified adjuvant and herbal adjuvant	India	[6]
Recombinant protein vaccine	Montanide adjuvant	Hungary	[16]
Recombinant protein vaccine	Single-walled carbon nanotubes (SWCNTs)	China	[17]
Recombinant protein vaccine	-	China	[18]
Recombinant protein vaccine	-	India	[19]
Recombinant protein vaccine	ISA 763 adjuvant	China	[20]
Recombinant protein vaccine	Freund's adjuvant	China	[21]
Recombinant protein vaccine	-	India	[22]
Recombinant protein vaccine	single-walled carbon nanotubes (SWCNTs)	China	[23]
Recombinant protein vaccine	Freund's adjuvant	China	[24]
Recombinant protein vaccine	ISA 763 adjuvant	China	[25]
Recombinant protein vaccine	PLGA Nanoparticle	India	[26]
DNA vaccine	single-walled carbon nanotubes (SWCNTs)	China	[27]
DNA vaccine	QCDCR adjuvant	USA	[28]
DNA vaccine	QCDCR adjuvant	USA	[29]

**Table 2:** Summary of experimented *A. hydrophila* fish vaccines in terms of antigenic entity used, fish models, route of administration, and reported efficacy.

Antigenic entity	Model fish specie	Route of administration	Reported efficacy	Reference
Lipopolysaccharide (LPS) and Outer Membrane Protein (OMP)	Grass Carp (Ctenopharyngodon idella)	Injected intraperitoneally	RPS 83.3, 72.2	[9]
Recombinant outer membrane protein R (rOmpR)	Rohu (Labeo rohita)	Injected intraperitoneally	RPS 52	[6]
Outer membrane protein (Omp-G)	eels (Anguilla anguilla)	injected intraperitoneally	RPS 50-75	[18]
Outer membrane proteins (OmpA1, Tdr, and TbpA)	Channel catfish (Ictalurus punctatus)	intraperitoneally (IP) injected	RPS 98.59, 95.59, and 47.89	[7]
Outer Membrane Protein (OMP)	Goldfish (Carassius auratus	Injected intraperitoneally	50%	[11]
Outer Membrane Protein (OMP)	American eel (Anguilla rostrata)	Injected intraperitoneally	RPS 50%	[21]
Outer membrane protein 48 (Omp48)	Rohu (Labeo rohita)	intramuscularly	RPS 69	[22]
Outer membrane proteins, (Aha1 and OmpW)	common carp	injected intraperitoneally	RPS 67 and 80	[19]
OmpW PLGA	Rohu (Labeo rohita)	orally administered	RPS 37.33- 79.99	[26]
Omp38	Chinese breams	intraperitoneally immunized	RPS 50.00-57.14	[20]
The iron-regulated outer membrane protein (OMP)	zebrafish,	injected intramuscularly	RPS 63.4-68.6	[24]
Live recombinant Lactococcus lactis vaccine expressing aerolysin genes D1 and D4	Tilapia (Oreochromis niloticus)	intraperitoneal injection oral feeding	RPS 55–82 RPS 70–100	[14]
Recombinant Aeromonas hydrophila vaccine (Aera)	grass carp	bath immunization	-	[23]
Recombinant protein aerA	Grass carp	bath immunization intramuscular injection	RPS 84.9 RPS 79.6	[17]
N-acyl Homoserine 1 Lactonase	Zebrafish	oral administration	-	[15]
Fimbrial Proteins (FimA, Fim, FimMrfG, and FimOM)	Channel Catfish	injected intraperitoneally	RPS 59.83, 95.41, 85.72, and 75.01	[13]
Maltoporin (46 kD)	European eel (Anguilla anguilla)	intraperitoneal injected	RPS 62.5-100	[12]
Glycoprotein-based native-subunit	Rainbow Trout (Oncorhynchus mykiss)	Immersion injected intraperitoneally	68.0%	[10]
Recombinant S-layer protein vaccine	common carp	vaccinated intraperitoneally	RPS 56-87	[16]
G-protein coupled receptor 18 (GPR18)	channel catfish	intraperitoneally injected	50-100%	[29]
Recombinant Hemolysin Co-regulated Protein (Hcp)	Common carp (Cyprinus carpio)	injected intraperitoneally	RPS 46.67	[25]
DNA vaccine (naked plasmid DNA)	grass carp	injected intramuscularly	RPS 68.9	[27]
Apolipoprotein A1 plasmid DNA	channel catfish	intraperitoneally injected	100%	[28]

**Table 3:** Synopsis of experimental setup of vaccine testing in the selected studies.

Study Reference	No. of fish used	No. of fish for immune response	No. of challenged fish	Day of challenge post vaccination
Gong et al. 2015	1920	720	540	28
Dash et al. 2014	870	90	180	56 and 140
Cao et al. 2012	720	-	-	-
Abdelhamed et al. 2016	700	70	-	21
Liu et al. 2016	640	240	240	21
Abdelhamed et al. 2017	500	80	420	21
Pridgeon & Klesius, 2013 [29]	420	-	420	2, 4, 14, 24, 28 and 48
Pridgeon & Klesius, 2013 [28]	420	-	420	2, 4, 14, 24, 30 and 48
Sun et al. 2010	400	-	80	35
Feng et al. 2017	360	72	90	28
Liu et al. 2015	300	-	-	-
Wang et al. 2013	300	300	90	45
Wang et al. 2015	300	144	90	45
Maiti et al. 2012	270	54	270	24
Thangaviji et al. 2012	240	-	180	30 and 60
Poobalane et al. 2010	240	-	240	35
Songlin et al. 2015	180	60	48	28
Wang et al. 2017	180	-	180	7 and 14
Anuradha et al. 2010	168	168	168	35
Dubey et al. 2016	160	160	120	50
Guan et al. 2010	156	36	120	28
Çiftci et al. 2016	100	100	100	21
Khushiramani et al. 2012	100	100	100	10
